# Targeting CNS Neural Mechanisms of Gait in Stroke Neurorehabilitation

**DOI:** 10.3390/brainsci12081055

**Published:** 2022-08-09

**Authors:** Jessica P. McCabe, Svetlana Pundik, Janis J. Daly

**Affiliations:** 1 Brain Plasticity and NeuroRecovery Laboratory, Cleveland VA Medical Center, Cleveland, OH 44106, USA; 2 Department of Neurology, Case Western Reserve University, Cleveland, OH 44016, USA; 3 Brain Rehabilitation Research Center, Malcom Randall VA Medical Center, Gainesville, FL 32608, USA; 4 Department of Physical Therapy, College of Public Health and Health Professions, University of Florida, Gainesville, FL 32608, USA

**Keywords:** gait, CNS, stroke, coordination, brain imaging, gait training

## Abstract

The central nervous system (CNS) control of human gait is complex, including descending cortical control, affective ascending neural pathways, interhemispheric communication, whole brain networks of functional connectivity, and neural interactions between the brain and spinal cord. Many important studies were conducted in the past, which administered gait training using externally targeted methods such as treadmill, weight support, over-ground gait coordination training, functional electrical stimulation, bracing, and walking aids. Though the phenomenon of CNS activity-dependent plasticity has served as a basis for more recently developed gait training methods, neurorehabilitation gait training has yet to be precisely focused and quantified according to the CNS source of gait control. Therefore, we offer the following hypotheses to the field: Hypothesis 1. Gait neurorehabilitation after stroke will move forward in important ways if research studies include brain structural and functional characteristics as measures of response to treatment. Hypothesis 2. Individuals with persistent gait dyscoordination after stroke will achieve greater recovery in response to interventions that incorporate the current and emerging knowledge of CNS function by directly engaging CNS plasticity and pairing it with peripherally directed, plasticity-based motor learning interventions. These hypotheses are justified by the increase in the study of neural control of motor function, with emerging research beginning to elucidate neural factors that drive recovery. Some are developing new measures of brain function. A number of groups have developed and are sharing sophisticated, curated databases containing brain images and brain signal data, as well as other types of measures and signal processing methods for data analysis. It will be to the great advantage of stroke survivors if the results of the current state-of-the-art and emerging neural function research can be applied to the development of new gait training interventions.

## 1. Background: Complexity of Gait Control

The central nervous system (CNS) controls the complex motor function of the gait pattern, coordinating interactions within the CNS, with the peripheral musculoskeletal system, and with feedback loops of the visual, vestibular, and proprioceptive systems. It is well-known that the CNS optimizes the energy required for human gait by engaging factors inherent in human body morphology, the force of gravity, rotational forces at the limb joints (torque or moment of force), and the physiological processes of muscle activations [1].

There is a growing body of literature detailing the brain and spinal cord neural structures and processes controlling the coordinated gait pattern. First, CNS structures of great importance to gait control include cortical areas (primary motor cortex, premotor cortex, supplementary motor cortex, somatosensory cortex, and prefrontal cortical areas), the basal ganglia, the thalamus, and the cerebellum [2]. Second, the intention to produce volitional movement begins in the prefrontal cortex, which signals the motor cortical areas to produce a motor signal [3]. Third, before being sent to the periphery, two refinements occur within the CNS. Projections from the cortex via the corticostriatal pathway deliver the motor signal to the basal ganglia [3]. The motor signal is modulated within the basal ganglia via the direct and indirect pathways; these pathways are further amplified by dopaminergic neurons in the substantia nigra via the nigrostriatal pathway [3]. The modulated motor signal is then relayed from the basal ganglia back to the cortex through the ventral anterior and ventral lateral thalamic nuclei. This signal modulation occurring within the basal ganglia is believed to influence the initiation of desired movement and inhibition of unwanted movement [3]. Additionally, the cortical motor signal is sent via cortico-pontine-cerebellar tracts to the cerebellum, which then integrates the motor signal with information from incoming afferent pathways of the visual, proprioceptive, and vestibular systems to refine the motor signal; the motor signal is then transmitted back from the cerebellum to the cortical motor regions through the contralateral thalamus via the dento-thalamic pathway, with the thalamus relaying the signal back to the cortical motor regions [3]. The refined motor signal is then sent via descending motor pathways, mainly via the corticospinal and reticulospinal pathways, to the spinal cord [3]. Fourth, within the spinal cord, the motor signal is transmitted to the lower motor neurons which, in turn, project to the muscle and activate muscle contractions [4]. Fifth, within the spinal cord, inter-neuronal networks play a role in the control of locomotion [4,5]. Evidence of a “central pattern generator” (CPG) within the spinal cord is evident across mammalian species [6]. The CPG in humans is believed to be at the level of the second lumbar vertebra (L2) in the posterior spinal cord [7] and is a rhythmogenic circuit kernel partially controlling locomotor movements, based on an open loop system that does not require sensory feedback [8]. Sixth, the mesencephalic locomotor region (MLR), which is thought to be located in the area of the pedunculopontine nucleus and the cuneiform nucleus, has been studied for possible influence in regulating locomotion via descending projections that stimulate reticulospinal neurons, which then activate the CPG neurons within the spinal cord [9]. Seventh, the vestibular system regulates balance via vestibulospinal reflexes that induce postural adjustments through muscle activations that control trunk and limb movements and positional control [8]. During the motor task of walking, postural and balance control are required [8] for sequentially challenging and changing dynamics with weight bearing on one limb, two limbs, and during weight transfer between limbs. Further detail regarding the normal neural gait control is available elsewhere [2,3].

With this evidence of the complex interactions of brain and spinal cord networks that control gait coordination, it is reasonable to consider the need for a paradigm shift in gait training methods in stroke neurorehabilitation. Indeed, it is critical to incorporate the neural control of normal gait, the disruption of neural pathways after stroke, and neuroplastic mechanisms of motor relearning into future research.

## 2. Hypotheses and Rationale 

### 2.1. Hypotheses

The current situation in the neurorehabilitation of gait coordination after stroke is that chronic gait dyscoordination does not respond adequately to standard practice methods; that is, the state-of-the-art research does not lead to the recovery of normal gait coordination. Therefore, in the interest of developing efficacious gait coordination training for stroke survivors, we would like to issue a call to our field by stating two hypotheses, as follows:

**Hypothesis 1.**  *Gait neurorehabilitation after stroke will move forward in important ways if research studies include brain structural and functional characteristics as measures of response to treatment.*

**Hypothesis 2.**  *Individuals with persistent gait dyscoordination after stroke will achieve greater recovery in response to interventions that incorporate the current and emerging knowledge of CNS function by directly engaging CNS plasticity and pairing it with peripherally directed, plasticity-based motor learning interventions.*

### 2.2. CNS Mechanisms of Gait Coordination Control after Stroke

#### 2.2.1. Drivers of CNS Plasticity

The phenomenon of neural plasticity entails the generation of neural structures and reorganization of neural function [10]. After a stroke, and in the presence of persistent gait dyscoordination, it is critical to engage neural plasticity in order to restore coordinated gait. A number of motor practice principles required for the neuroplastic response have been identified, including the following: practicing as close-to-normal joint movement coordination as possible [11,12,13,14] with continual progression toward normal as the ability improves [15,16]; high repetition of the desired coordinated joint movements [17,18,19]; attention focused on the motor task at hand [20], training specificity [21,22,23], which in this case entails the practice of coordination gait components; and awareness and feedback [23,24].

#### 2.2.2. Structures Critical to Recovery

Longitudinal neuroimaging studies spanning the recovery period after stroke are beginning to inform neurorehabilitation research by identifying the CNS structures critical to recovery. For example, damage to the ipsilesional corticospinal tract (CST) has a devastating impact on motor control [25]. In fact, the integrity of the ipsilesional CST has been successful in predicting the recovery of gait function [25,26]. Other descending tracts also play roles in gait control, and recent work has suggested that, in addition to ipsilesional CST, the integrity of the ipsilesional cortico-reticulospinal tract and contralesional superior cerebellar peduncle tracts are predictors of walking performance, even at 2 years post-stroke [26].

#### 2.2.3. Neural Function Influencing Recovery

Some scientists have identified a post-stroke abnormal balance of cortical excitability, with elevated contralesional signal and diminished ipsilesional excitability [27,28]. This abnormal balance could create an elevation of inhibitory signals arising from the contralesional hemisphere, which may normally be opposed by the ipsilesional hemisphere. With neural damage in the ipsilesional hemisphere, the contralesional inhibitory signals may further weaken muscle activation processes. This transcallosal interaction after stroke is complex and is likely dependent on the degree of damage to the corticospinal tract [29].

Some have noted that abnormal neural activity post-stroke can result in abnormal co-contractions of the agonist (prime mover) and antagonist muscles at a given lower-limb joint during walking [30,31,32]. This motor dyscoordination and abnormal co-contractions produce swing phase deficits including the inability to flex the hip, knee, and ankle of the involved limb, resulting in abnormal timing and limited movement excursion (e.g., [33]). Abnormal co-contractions, during the stance phase, result in the inability to coordinate the hip, knee, and ankle joint movements for dynamic balance control across the subphases of stance, including the absence of the 15 degrees of knee flexion that normally cushions the lower-limb joints and reduces the energy-costly vertical center of mass (COM) oscillations [34]. Further, these co-contractions impose the compensatory strategy of a slowed gait speed, preventing the normal conservation of mechanical energies (kinetic and potential energies) within and between the thigh and shank, which normally produce knee flexion during the swing phase [35], without muscle activations [34,35]. In this way, the post-stroke abnormal balance of neural signals produces a cascade of physiological processes resulting in the well-known post-stroke, dyscoordinated gait patterns [36,37].

#### 2.2.4. Measuring Brain Activity during Gait after Stroke

##### Methods

Researchers have begun to characterize brain signals during walking after stroke. Methods for non-invasive brain signal acquisition include functional near-infrared spectroscopy (fNIRS, e.g., [38]; stroke and older adults); electroencephalography (EEG, e.g., [39]); and positron emission tomography with fluorodeoxyglucose (PET/FDG, e.g., [40]). fNIRS is an optical imaging method that quantifies change in blood (de)oxyhemoglobin (Hb) concentrations in the brain by characterizing the absorption spectra of Hb in the near-infrared range. The fNIRS signal is used to infer the changes in neural activity; its capability is limited to measurements within 2.5 cm of the skull surface. Its advantage is its ability to infer the regions of activation during walking. The EEG is a method of measuring electrical activity acquired at the scalp surface, which is a manifestation of electrical activity in the brain. An advantage of EEG measures compared with other imaging methods is the relatively more precise timing of the EEG signal characteristics, such as signal activation onset latencies or timing of waveform frequency changes. A disadvantage of the EEG methods is the relative lack of precision in the location of the signal. Methods to overcome these disadvantages include high-density EEG electrode arrays, source localization calculations [41], and combined imaging methods such as simultaneous fNIRS/EEG collection [39]. PET/FDG measures the presence of glucose, which is used to infer regions of elevated brain neuronal activity.

##### Differential CNS Activation Patterns across Whole-Body Gait Tasks after Stroke

A number of researchers have characterized stroke gait using the fNIRS/EEG methods. A recent review categorized and described the results from multiple studies on stroke gait; they categorized the published studies according to particular motor aspects of walking such as “initiating walking”, “accelerating walking speed”, “steady-state walking”, and “asymmetrical gait tasks” [39,40]. Together, the studies showed agreement that, during gait initiation and acceleration, there were symmetrical increases in the brain signal activity in regions controlling motor planning and motor performance, such as the prefrontal cortex (PFC), the supplementary motor area (SMA), and the sensory–motor cortex (SMC). A number of studies focused on steady-state walking, finding an elevation of contralesional activation in regions serving motor performance and sensory integration, such as the SMC and parietal regions; this relative elevation of signal magnitude appeared to be greater during slower-speed walking and varied according to the gait coordination ability. Some studies focused on and reported elevated ipsilesional brain signals during complex walking tasks, for which stroke survivors exhibited more elevated brain signals compared with healthy young and older adults [42].

#### 2.2.5. Measuring the Change in Gait-Related Brain Activity in Response to Treatment

A few studies describe walking-related brain activity change in response to gait training. In one study, there was a correlation of post-gait-training change in the Berg balance scale and ipsilesional EEG-based event-related synchronization (ERS) in the alpha and low-beta bands (r = −0.52, *p* = 0.039; r = −0.52, *p* = 0.040, respectively; [43]. Second, a group reported a reduction in prefrontal executive function resources according to the fNIRS signal following therapy [44]. Third, researchers studied EEG-based signal connectivity in the walking-related frequency (24–40 Hz) over the frontal–central–parietal areas during walking. They studied two different interventions, finding greater change in brain function manifested by higher levels of cortico-cortical and cortico-muscular connectivity in response to the more challenging training [5]. In addition, functional connectivity (e.g., [45]) is one measure of recent interest with regard to changes in response to treatment. In a summary review, Lim et al. [42] reported that studies are showing improvement toward normal in response to gait training and in association with gait improvement, according to mitigation in abnormal signal elevation and asymmetrical neural activations. However, for those with severe stroke, studies reported persistent abnormal signal elevation.

#### 2.2.6. Non-Invasive Brain Stimulation as an Intervention for Gait Dyscoordination after Stroke

Two of the available non-invasive brain stimulation methods are transcranial magnetic stimulation (TMS) and transcranial direct current stimulation (tDCS), each with advantages and disadvantages. One reasonable approach for a brain stimulation paradigm to enhance the activity-dependent plasticity during motor-learning-based gait therapy is by the facilitation of the ipsilesional motor pathways while inhibiting the contralesional outflow pathways [46,47]. In fact, studies in stroke survivors showed that brain stimulation could not only modulate the balance of excitability between the two brain hemispheres but could also produce some associated functional recovery [48,49]. Additionally, better motor outcomes are most likely to occur when brain stimulation is administered with peripherally directed, task-specific activity versus brain stimulation alone [50]. Others have published preliminary work on adding brain stimulation methods to the treatment of the lower limb after stroke [49,51,52,53,54,55], and the results are somewhat promising [49,51,52,53,54,55]. In chronic stroke, one recent study demonstrated the feasibility of administering targeted tDCS brain stimulation during stance phase walking training, designed and framed within neuroplastic motor learning principles [56]. 

Taken together, this rapidly growing body of work demonstrates both exciting emerging discoveries and the need for more sophisticated neural imaging and neural stimulation tools, treatment methods, and signal processing analyses. 

## 3. Future Work

Going forward, it is important to consider and incorporate the work that has been completed in characterizing disruption in neural structures, pathways, and functional activation during dyscoordinated gait after stroke. Meaningful results will be ensured through the use of more specific measures of CNS function, targeting CNS function in treatment methods, and the use of carefully designed, valid study design.

### 3.1. Potential Directions of Inquiry

#### 3.1.1. Moving beyond the Current Milieu

On behalf of stroke survivors and gait training research, there is a large body of traditional literature that entails peripherally directed interventions, including treadmill training with and without body-weight support [57], robotics, functional electrical stimulation (FES; [16,58]), bracing, strength training [59], and over-ground gait training (e.g., [60]). Many of these studies struggled to show any change in the actual gait coordination control. With promising results, some clinician scientists engaged the published, plasticity-based motor learning principles in gait training (e.g., [33,61]) and administered them accordingly in long-duration protocols [62,63]. Though promising, many study participants did not recover normal gait coordination. A limitation of these traditional, peripherally directed interventions is that many have not included measures of CNS change in response to treatment, nor treatment targeted to specific CNS function or pathways. To bring gait training research into contemporary times, it is important to measure CNS function and target CNS function in treatment methods.

#### 3.1.2. Brain Activity Patterns for Gait Characterization

The categorization of gait “tasks” by Lim et al. (2021) [42] is important because it illustrates the differences in brain activation patterns for the “high-level” gait components such as gait initiation, steady-state walking, and asymmetrical gait tasks. These can be considered gait whole-body movement tasks, within the force-of-gravity environment and the associated biomechanical principles governing whole-body movement. This is important emerging information, and at the same time, it is critical to be more specific in identifying the brain control of the gait coordination components of both stance and swing phases, as well as dynamic balance control.

#### 3.1.3. CNS Control of Balance and Lower-Limb-Coordinated Movement during Walking

Moving beyond whole-body movements as described above, there is a need to now identify and measure CNS function that controls two of the basic components of gait coordination. The first is the CNS control of balance coordination (single limb, double limb, and weight transfer) during walking (e.g., [64]). Understandably, balance during walking is less understood versus static standing balance or balance during gait initiation. However, existing information provides several sources of control and mechanisms that are employed to prevent imbalance or a fall in healthy adults, including the following: the visual system [65]; the neural modulating effect of changing body configuration and balance requirements across the gait cycle [66,67]; foot placement mechanisms [68,69,70]; lateral ankle roll mechanism to complement foot placement control [71,72]; and systematic changes in the modulation of the ankle plantar-/dorsiflexion angle and associated musculature near the end of the double stance phase [73]. However, there is little information as to the neural control of these identified muscle-driven kinematic mechanisms of balance control during walking. The existence of abnormal intraspinal processing has been demonstrated and implicated (e.g., [74,75]). However, the source of this pathophysiology is not well-understood. Some have reported indirect evidence of supraspinal origins of spasticity, occurring due to an imbalance of descending inhibitory and facilitatory signals that normally would regulate spinal stretch reflexes (e.g., [76]). Additionally, the hyperexcitability of the reticulospinal (RST) is implicated in balance deficit after stroke [76], but direct measures are needed to rule out other sources of pathophysiology [76]. From a clinical intervention perspective, information is emerging that characterizes changes in brain signals associated with improved dynamic balance measures (e.g., the timed-up-and-go test, [77]). Taken together, these initial discoveries provide new directions of inquiry.

A second basic component of gait coordination that requires study is the CNS control of limb movement through volitional muscle activation coordination across each joint of each limb and across the joint within a given limb. One less explored and potentially fruitful direction of inquiry is the use of CNS neural feedback training for lower-limb coordination recovery. The traditional feedback in motor training includes studies using electromyographic (EMG) signal feedback, with enough promise to deserve consideration in current work [78], perhaps in combination with CNS measures and CNS-targeted interventions. More recent studies have attempted to employ brain signals in neural feedback systems post-stroke, for both upper-limb motor training (brain–computer interface (BCI) or brain–machine interface (BMI, e.g., [79])) and lower-limb motor training [80]. However, more precise neural signals are needed in order to effectively guide motor recovery. One direction of inquiry could be to employ the brain neural signals to enhance the agonist muscle activation and mitigate the abnormal antagonist muscle activation. An attempt was made to differentiate the fMRI voxel activity in the primary motor cortex (PMC; (M1)) during simple active joint flexion versus simple joint extension [81]; the reported difficulty was that the active voxels during flexion and extension at the given joint were adjacent neighbors in the brain, and further, some voxels were active during both flexion and extension movements. Due to both the overlap and close proximity of active neurons for single joint extension and flexion, it was not possible with those study methods to differentiate the respective signals of joint extension versus flexion, for use in future training paradigms for stroke survivors [81]. In the future, in more sophisticated studies to differentiate the volume of activation for joint flexion versus extension, researchers could consider greater imaging precision (smaller voxel size) and 7T fMRI) [81]. Though fNIRS is a less precise imaging method compared with fMRI, one study used the fNIRS signal in brain neural feedback. In a randomized controlled trial, one group was administered the fNIRS-based brain signal feedback during motor training, and the results showed that only the feedback intervention group showed significantly increased imagery-related SMA activation and enhancement of resting-state connectivity between the SMA and ventrolateral premotor area, along with differential improvement in the timed-up-and-go test [77]. Some are developing new measures of brain function (e.g., [82]). More sophisticated imaging methods with more precision and greater resolution would importantly enhance the potential use of brain neural feedback training [79]. A unique advantage of biofeedback, in general, is that it uses and seeks to enhance neural function, in contrast with some gait interventions that externally impose a stimulus, such as robotics, FES, or brain stimulation.

#### 3.1.4. Large Database of CNS Control of Gait and Recovery of Gait Coordination

The scope of future undertakings is complex and will require many researchers working both independently and together. There is an impressive and encouraging current increase in the study of the neural control of motor function by independent research groups. At the same time, a number of groups have developed and are sharing sophisticated, curated databases containing brain images and brain signal data (e.g., [83]), as well as other types of measures and signal processing methods for data analysis (e.g., [84]). These large-scale, cooperative endeavors are critical in advancing the study of gait coordination recovery.

### 3.2. Study Design Issues

In testing the two proposed hypotheses, there are study design issues to consider for future work, namely the development and use of measures, the construction and content of interventions, and basic study design.

#### 3.2.1. Variables with the Potential to Confound or Diminish the Results for any Given Study

For efficacy studies of new interventions, it is critical to exclude acute and subacute stroke survivors if their presence confounds the study with spontaneous recovery (<6 months post-stroke); that is, it is well-known that stroke survivors can improve without treatment in the domains of impairment and function (e.g., [85,86,87]) up to 6 months after the stroke. However, there is a need for efficacious interventions in the acute and subacute phases, especially for more severely impaired stroke survivors. Indeed, it is obvious that there is a need to identify the brain neural intervention to restore normal gait coordination for severely impaired stroke survivors; once that efficacious intervention is identified, then it would, of course, be tested and offered in the acute/subacute phases after stroke. In researching the topic of acute/subacute interventions, it is reasonable to consider the unique aspects of brain plasticity that could be engaged during the acute/subacute timeframes. The currently available evidence points to the need for and benefit of more intense and longer duration intervention in the clinical care of acute and subacute stroke survivors e.g., [62,63].

It is important to account for measurement error, through the use of the minimally detectable threshold for change (MDC) for each given measure [88,89,90]. Currently, some studies are reporting a change that is actually only in the range of measurement error, not real change; this is an unfortunate and misleading practice that obfuscates the accuracy of treatment response. The minimal clinically important difference (MCID) is derived to estimate the change in score for the given measure that would be required for a clinically important and meaningful improvement [91,92,93,94]; in order to incorporate real-life meaning into study results, the MCID should be incorporated into study design and results reporting.

#### 3.2.2. Accurate Interpretation of Results in Response to Gait Training Intervention

Others have described the need for “domain-specific” measures in stroke research [95], and this is certainly true in gait coordination research. In fact, for example, there are serious consequences of inaccuracy and error in results reporting [96] if gait speed is used as the primary outcome measure without supporting impairment measures such as gait coordination and balance. We note that experience in the field has illustrated that a stroke survivor can increase the speed of his/her dyscoordinated and unsafe gait, at will, without changing the balance deficit or dyscoordination. “Increased gait speed” does not necessarily provide any information as to whether the gait pattern actually improved in coordination and balance or fall prevention. Rather, to accurately interpret any change in gait speed, one must also analyze changes in balance and coordination. Though clinical facilities do not provide the motion-capture technology analysis of gait kinematics, well-respected observational clinical measures of gait coordination are available [97,98,99,100], in some cases requiring only a video camera or a phone camera, and stop-frame viewing capability. Further, our standard measures of balance control can have a ceiling effect, whereas more precise measures, such as the center of pressure (COP) measures, can be more informative and sensitive to treatment response [101]. With credible measures of gait coordination and balance available, we can usefully recall that “reduced” gait speed is not a gait deficit; rather, it is a compensatory strategy adopted by the stroke survivor because of weakness, abnormal muscle co-contractions, proprioceptive disruption, and/or lack of balance control. If gait speed is the only measure of focus, there is the potential to miss the actual improved coordinated components of gait, precluding further study of a potentially efficacious intervention. Additionally, there are instances in which improvement, in response to a new treatment, could occur in important ways without any change in gait speed. Examples include walking endurance; balance; limb coordination for functional mobility; reduction in falls; and mitigation of fear of falling.

#### 3.2.3. Use Knowledge of the Array of Pathologies and Impairments after Stroke to Construct Valid Hypotheses and Intervention Content, and Valid Outcome Measures

For some purposes in developing new interventions, a tight study design without confounds is the obvious choice. However, caution is needed in some respects because stroke survivors present with an array of impairments. In that case, according to simple logic, it is pointless to expect functional change from an intervention targeted to only one impairment, when multiple impairments preclude normal function. Rather, in contemporary clinical science studies, there is an important place for studies in which there is an accurate evaluation of each individual and application of a combination of interventions designed to target each existing impairment preventing gait coordination recovery (e.g., [33,62,63,102]). That type of study design procedure will necessarily bring difficulties in controlling moderating variables, but it will bring neurorehabilitation research into the contemporary world of “precision medicine” (customized to the patient), that is, precision neurorehabilitation research.

#### 3.2.4. Encourage Inter-Disciplinary Teams and Consultants, including Neurorehabilitation Specialists, Engineers, Biomechanistst, and Physicists

It is important to incorporate the existing information from neuroscience, biomechanics, engineering, biophysics, and other relevant fields to prevent the repetition of prior failed methods in both research study design and clinical practice. For example, it is well-known that active, purposeful movements are required in order to reap potential neuroplasticity-based recovery of motor control. However, a number of robotics studies in the past appeared to assume that robotically imposed movements would produce motor recovery. By the same token, a number of studies were published in the past that appeared to assume that functional electrical stimulation (FES) systems could be simply applied for practice sessions, without any accompanying attention from the user. These studies failed to produce meaningful motor recovery and, as such, support a call to evaluate technologies in terms of capabilities in delivering the motor practice paradigms that are efficacious, based on known motor learning neuroplasticity phenomena [103,104]. In fact, the ideal situation is that a neurorehabilitation expert would serve as a formal member of technology design teams.

## 4. Summary

The evidence now exists for gait interventions after stroke to be elevated to a new level of scholarly excellence by targeting CNS structures, pathways, and mechanisms. The extant literature supports the need for more precise methods of non-invasive CNS signal measurement, analysis, and brain stimulation techniques. It will be to the great advantage of stroke survivors if the results of the emerging and current state-of-the-art neural function research can be applied to the development of new gait training interventions.

## Data Availability

Not applicable.
